# Cooperative Role of Mixed Solvent in the Evaporation-Induced Self-Assembly of Polypeptoid Nanocrystals

**DOI:** 10.1021/acsanm.5c01381

**Published:** 2025-06-16

**Authors:** Xubo Luo, Fabrice Roncoroni, Tianyi Yu, Nan K. Li, Ronald N. Zuckermann, Xi Jiang, Nitash P. Balsara, David Prendergast

**Affiliations:** † Materials Sciences Division, 1666 Lawrence Berkeley National Laboratory, Berkeley, California 94720, United States; ‡ Molecular Foundry, Lawrence Berkeley National Laboratory, Berkeley, California 94720, United States; § Department of Chemical and Biomolecular Engineering, 1438 University of California, Berkeley, California 94720, United States

**Keywords:** polypeptoid, self-assembly, nanocrystals, solvent mixtures, evaporation-induced, surface adsorption, preferential solvation, molecular dynamics

## Abstract

Peptoids, or polypeptoids, are biomimetic polymers that can self-assemble into nanocrystals for biomedical and biotechnological applications. Polypeptoid nanocrystals can be prepared by evaporation-induced self-assembly, but the roles of solvent components for this process have long been overlooked at the molecular level, leaving a tunable parameter for improving self-assembly protocols. This work utilized molecular dynamics simulations to study the effects of water and the commonly used tetrahydrofuran (THF) on the assembly of nanosheets from molecules of acetylated diblock polypeptoid, poly­(*N*-decylglycine)-*b*-poly­(*N*-2-(2-(2-methoxyethoxy)­ethoxy) ethylglycine), abbreviated as Ac-Ndc_10_-Nte_10_. To probe the stages of self-assembly, isolated molecules and preassembled nanofibers/nanosheets were simulated in pure THF, water, and their mixtures, respectively. The assembly energies show that the THF/water mixture has a greater tendency to form nanosheets than pure water. In a THF/water mixture, polypeptoids were found more uncoiled in isolated states, less compact in disordered agglomerates, and with reduced requirement for the Nte block to cover hydrophobic Ndc surfaces in the nanocrystals. Mixed solvent is vital to initiating self-assembly, as THF assists in the opening of coiled polypeptoid molecules, while water provides the thermodynamics to aggregate and ultimately form nanocrystals. To obtain wider nanosheets, it is recommended that some THF be maintained in the aqueous solvent before it becomes exhausted by evaporation. Near the nanosheet surface, the THF concentration is higher than that in bulk solution (3–4 times in 4 M THF/water). The strong adsorption of THF indicates the self-assembly in a *de facto* mixed solvent. These results are expected to guide the refinement of evaporation-induced self-assembly protocols for polypeptoid nanocrystals.

## Graphical abstract

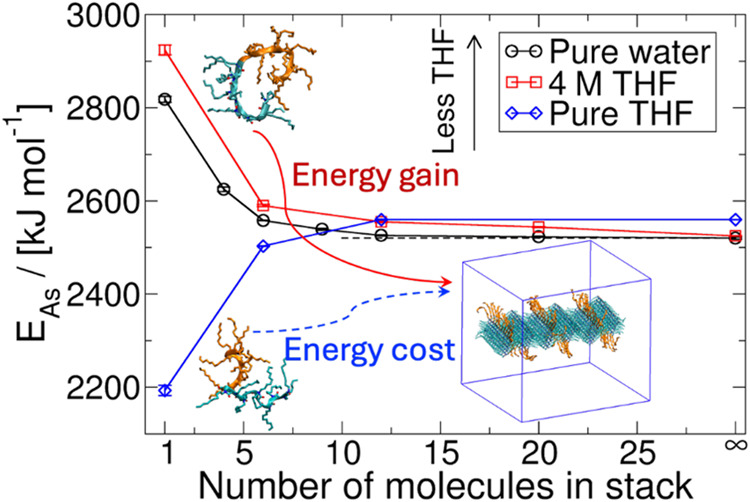

## Introduction

Polypeptoids make up a class of synthetic protein mimetics, which consist of isomers of natural amino acids as the monomers. The side chain in each monomer is tethered to the backbone nitrogen instead of the α-carbon.^1,2^ The particular chemical structure of polypeptoids, as distinct from polypeptides, lacks the hydrogen bond donor of the amide (N–H) in their backbones, which allows intermolecular nonbonding forces (*viz*., electrostatics, dispersion) to dominate their self-assembly behaviors.^3^ With a wide library of side chains available for attachment at the backbone N, the sequence of a polypeptoid molecule is programmable, which has been exploited for promising applications in biomedicine and biotechnology.^4–8^ Polypeptoids can be precisely controlled by tuning the monomer sequences or blocks to self-assemble as nanofibers, nanosheets, and nanobrushes.^2,9–13^ These nanostructures typically have highly ordered structures at the subnanometer scale, as confirmed by cryo-electron microscopy (cryo-EM) images and X-ray diffraction, which suggest that the backbones and side chains are well-aligned and packed in a layer-by-layer arrangement inside the nanocrystals.^12,14–16^

To prepare polypeptoid nanocrystals, evaporation-induced self-assembly is a commonly used method.^17^ A typical experiment fully dissolves the synthesized polypeptoid in a pure organic solvent, adding an equal amount of water, and allowing slow evaporation of the organic solvent component (which can take days to weeks) to allow the assembly.^8,11,18–22^ Despite the lack of direct experimental evidence, such as time-resolved imaging, it has been proposed that the assembly mechanism is hierarchical, as shown in Figure 1a.^2,23,24^ Polypeptoid molecules would first aggregate into disordered agglomerates, undergo backbone stretching/straightening and rearrangement into highly ordered molecular packings, and finally grow or combine into one-dimensional nanofibers and two-dimensional nanosheets. Recently, the existence of disordered precursor was observed by Lee et al. using cryo-EM at predetermined time intervals during self-assembly.^25^ Similarly, the early stage of peptoid nanosphere was revealed in a peptoid-templated silification of a nanoribbon.^26^ This evidence further supported the proposed multistep mechanism of nanosheet self-assembly. Presumably, the assembly is correlated with the decrease in the level of organic solvent due to the preferential evaporation, which approaches zero over time. Based on the proposed mechanism, precise control of solvent evaporation should assist the formation of high-quality nanocrystals with desired shapes and dimensions. However, laboratory protocols for polypeptoid evaporation-induced self-assembly are usually rudimentary, which are limited to unattended evaporation of the organic component at room or refrigerated temperatures.^9,11,17,18,21^ The ignorance of well-controlled evaporation might be due to the unawareness of the critical role played by solvent components at the molecular level. With fundamental knowledge of solvent effects, there could be considerable potential to improve and refine the evaporation-induced protocol for polypeptoid self-assembly and crystallization techniques.

**Figure 1:**
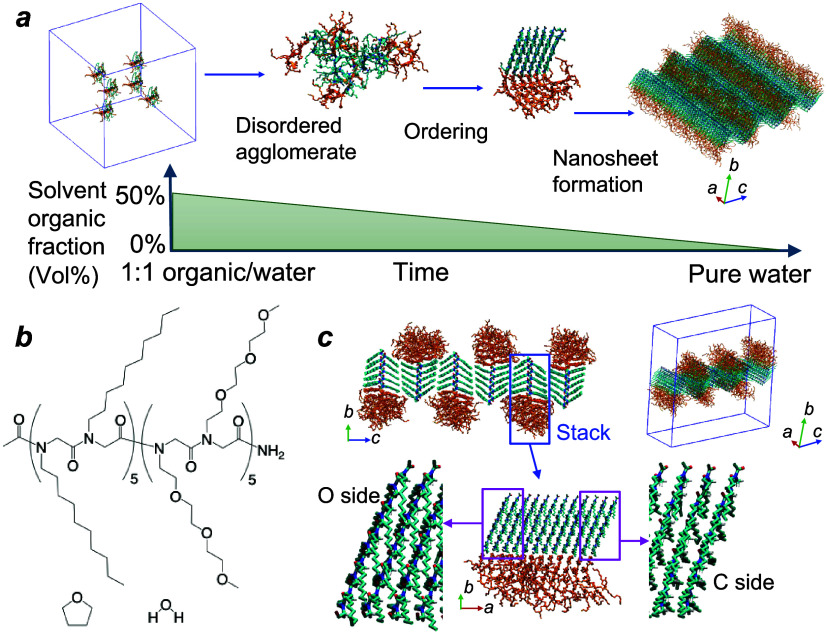
Chemical structure, nanosheet, and hypothesis of hierarchical self-assembly. (a) Hypothetical mechanism proposing that disordered agglomerates and ordered nanosheets are formed while the concentration of organic compounds in the solvent continuously decreases. Reproduced from ref 24. Copyright 2022 American Chemical Society. (b) Chemical structure of diblock polypeptoid and THF/water solvent. (c) Preassembled nanosheet consisting of stacks with layer-to-layer molecular packing. In (a) and (c), cyan: Ndc; orange: Nte. In each stack, all −CH_2_ backbone groups face one side (C-side) and all −C=O groups face the other side (O-side).

To date, there is very limited published research on the influence of solvent composition on the self-assembly of polypeptoids due to the challenge of real-time *in situ* measurements in position space that might connect time-evolving assembly with solvent concentration. Furthermore, there is little theoretical guidance on whether precise control of solvent evaporation is necessary. In recent work, Zhao et al. adopted a computational approach to investigate the effects of solvent.^24^ Their work simulated the free energies for attaching/detaching an additional peptoid molecule to an existing nanostructure in various organic/water solvents, which provides one of the few theoretical studies addressing the role of the solvent. Additionally, an experimental study has reported that adding urea to aqueous solvent promoted a more ordered crystalline phase of polypeptoid nanofibers, indicating the positive impact of employing a water solvent with an organic additive.^18,27^

This work aims to reveal how the solvent mixture can be an important factor in the entire process of evaporation-induced self-assembly. We employ molecular dynamics (MD) simulations to investigate the self-assembly of the acetylated diblock polypeptoid, poly­(*N*-decylglycine)-*b*-poly­(*N*-2-(2-(2-methoxyethoxy)­ethoxy) ethylglycine), abbreviated as Ac-Ndc_10_-Nte_10_ (Figure 1b). This diblock polypeptoid is well known for its nanosheets with highly ordered internal structure, as confirmed by processed cryo-EM images.^11,19^ Due to the extremely long time required for self-assembly (days to weeks), our prior MD simulations adopted preassembled structures as initial states inspired by analysis of cryo-EM imaging.^28^ Following equilibration of these structures, and consistent with experimental characterization, the relaxed nanosheets consisted of straight/extended Ndc blocks in the crystalline phase, where the Ndc backbone adopted an all-*cis* ∑-strand (aligned along the crystalline ***b*** axis).^11^ The molecules pack into molecular stacks (along the crystalline ***a*** axis) with backbone methyl groups oriented to one side and backbone carbonyl groups to the other side of the growth direction, which were named “C-side” and “O-side,” respectively, as shown in Figure 1c. These stacks can then combine with others side by side through dispersion interactions at their decyl side-chain tips (aligned along the crystalline ***c*** axis) to form nanofibers and nanosheets as the stacks increase in length. By contrast, molecular dynamics revealed that a single Ac-Ndc_10_-Nte_10_ chain exhibited a curved/coiled Ndc backbone covered by less hydrophobic Nte in pure water (Figure S1). By simulating various hypothetical stages of nanocrystal growth, these simulations demonstrated the thermodynamic driving forces behind polypeptoid self-assembly in pure water, validating the hierarchical self-assembly hypothesis (due to decreasing order of magnitude for the potential energy differences between initial short stack formation vs the combination of stacks into extended fibers and ultimately ordered 2D nanosheets). Using the same approach, the present work extends the investigation to different solvent environments. Isolated molecules and preassembled structures were solvated in tetrahydrofuran (THF)/water mixtures at different ratios to mimic the experimental evaporation-induced process, as in Table 1, where the mixtures of ∼50% wt, 4 M, and 5% vol. correspond to the beginning of evaporation,^11,16,19^ the level that organic solvent improved the quality of peptoid nanocrystals,^18^ and the small amount of THF at the end of evaporation in some previous experiments,^18,25^ respectively. We focus on evaluating the energetics to illustrate the thermodynamics underlying self-assembly. Analyses of spontaneous aggregation and single-chain backbones show the effects of different solvent mixture components, corresponding to the tendencies of agglomeration and backbone straightening. Furthermore, selective solvation and the adsorption of THF are also discussed to unveil the local environment near the nanomaterials. Our simulations serve to deepen our understanding of polypeptoid self-assembly, to inspire time-dependent studies of the assembly process, and to provide insight into the molecular design of these materials for the purposes of exacting precise control over desirable nanocrystal assemblies.

**Table 1:** THF/Water Molar Ratio in Simulation

system name	pure THF	∼50% wt	4 M	5% vol	pure water
THF/water	∞	0.25	0.072	0.012	0

## Methodology

### Classical MD Simulation for Preassembled Nanostructures

The model was identical to our prior publications.^18,28^ A CgenFF-based force field extended by Weiser and Santiso for peptoid was used for the backbones of acetylated Ndc_10_-Nte_10_,^29^ and the side chains and THF molecules were modeled as the ligands in the standard CGenFF.^30^ The TIP3P model was adopted for water. MD simulations were carried out using GROMACS (version 2022.5) with GPU acceleration.^31^ The cutoff distance was set to 12 Å for both van der Waals and electrostatic forces, and the particle-mesh Ewald summation was used for the long-range interactions for the latter. A 2 fs time step was selected with the utilization of the LINCS algorithm to fix the chemical bonds involving hydrogen.^32^ Preassembled nanofibers and nanosheets were built with packed molecular stacks of well-aligned Ndc backbones and side chains in an all-cis conformation. The stacks were connected at the side chain tips along one dimension of a box of periodic boundary conditions, which mimics the long nanofiber. To study the effect of nanofiber growth, this work performed a series of simulations, which had 6, 12, and 20 well-packed molecules in each stack to increase the width of the nanofiber. For the nanosheets, all 12-molecule stacks fully filled the second dimension of the periodic boundary box and were thus equivalent to a two-dimensional nanosheet. A tilt angle of ∼65° was adopted based on the test of our prior work for nanosheets.^28^ With Ndc blocks fixed using a spring constant of 1000 kJ/mol/nm, a 10 ns Langevin dynamics in vacuum was first conducted to quickly obtain the amorphous Nte blocks. The Ndc block was then released to free, and the entire nanostructure was solvated with the desired solvent of a THF/water mixture. The simulation box was subsequently equilibrated for 90 ns in the NpT ensemble at 300 K and ambient pressure. The temperature was controlled by a Bussi thermostat,^33^ and the pressure was maintained at 1 atm with a Berendsen barostat, followed by Parrinello–Rahman barostat.^34,35^ After that, several consecutive runs of 50 ns each were performed, while the potential energy was under careful monitoring. The simulation was considered complete when the potential energy became stable, and the last three trajectories of 150 ns in total were saved for analyses. The simulated structures were visualized using the Visual Molecular Dynamics (VMD) package.^36^

### Replica Exchange Simulation for Isolated Molecules

To enhance the sampling of conformation, replica exchange with solute tempering (REST2) was performed for an isolated polypeptoid molecule in explicit solvent.^37^ A single polypeptoid chain was fully solvated to run a classical MD simulation at 300 K and ambient temperature in NpT for 5 ns, aiming at the equilibrated density. REST2 was performed based on the correct density in an NVT ensemble with a Bussi thermostat at 300 K. The temperature ladder was built to cover 300 to 600 K, which was achieved by scaling the nonbonded and dihedral parameters for polypeptoid using PLUMED (version 2.7.2).^38^ The exchange rate was ensured to be >25% for all replicas. GROMACS 2021.5, patched with PLUMED 2.7.2, was employed. All other settings were the same as the simulations of preassembled structures, as described above. A trajectory of 60 ns was collected for each solvent type.

### Dimensionality Reduction and Clustering

The uniform manifold approximation and projection (UMAP) dimensionality reduction technique was adopted to embed the multidimensional structural data of peptoid atom coordinates into a two-dimensional space, which was conducted using hyperparameters of 15 for the number of neighbors and 0.0 for the minimal distance of close points.^39^ The training data included 3001 conformations from the REST2 simulation for an isolated molecule, 306 polypeptoid molecules from the C-side of a six-layer nanofiber, and 144 molecules from nanosheets, which are repeatedly collected for pure water, 5% THF/water, 4 M THF/water, and pure THF. Additionally, 202 conformations were collected from the C-side and the O-side of a dimer stack, respectively, for pure water and 4 M THF/water mixture. Only the heavy atoms of the Ndc backbones were considered, and the Cartesian coordinates were then converted to the internal distance matrices (distances to the first three atoms) to maintain rotational and translational invariance, which were used to train the UMAP transformer and generate a two-dimensional embedding of the data. This approach preserves the topology of the original data, being capable of revealing correlations hidden in complex data sets, and provides a more facile space for clustering algorithms. Proximity in the reduced (2D) UMAP space implies proximity in the original higher-dimensional space and hence conformational similarity. It is acknowledged that the obtained UMAP could be different if the training set was varied or alternatively organized, but this difference should not affect the comparison of different solvents. After obtaining the transformed UMAP, the algorithm of hierarchical density-based spatial clustering of applications with noise (HDBSCAN) was employed to cluster the data points of isolated molecules in order to identify the conformational groups that were closest to the nanofibers and nanosheets.^40^ The minimal cluster size was set to 10, the minimal neighbors for a point to be a core point was set to 3, and the distance threshold to merge two clusters was set to 0.2. For the yielded clusters, the average backbone conformation was reconstructed from the internal distance and displayed with the Atomic Simulation Environment (ASE).^41^ A brief schematic diagram of this workflow can be found in the Supporting Information (Figure S2).

### Umbrella Sampling for THF Adsorption

Umbrella sampling was performed for 4 M THF/water and pure water with one THF molecule for the nanosheet system, where the polypeptoid nanostructure was truncated to two stacks. GROMACS 2021.5, patched with PLUMED 2.7.2, was used.^31,38^ One THF molecule was selected as the sampling molecule, and the collective variable was chosen as the minimal distance from the center of mass of this THF to the nearest atom of the Ndc block. This distance was calculated with the switching function to ensure the continuity: $s=\beta /\log\sum_{i}\exp\left(\frac{\beta }{s_{i}}\right)$ , where *s* is the calculated minimal distance, *s*_*i*_ is the distance of THF to an arbitrary Ndc atom, and the weight β was set to 100. The pulling of THF started from adsorption at the decyl side chain. The increment was set to 0.05 nm, and a total of 81 sampling windows were constructed, covering the range of 0.35 to 4.4 nm. The force constant was set to 3000 kJ/mol/nm^2^, and 20 ns of simulation was completed for each sampling window in an NpT ensemble at 300 K and ambient pressure. Other settings are the same as the simulations of preassembled structures. The potential mean force, equivalent to Δ*G*, was calculated using the WHAM program (version 2.0.11).^42^

## Results and Discussion

### Energetics for Nanomaterials in Different Solvents

Figure 2a plots the assembly energy profile for different nanostructures in various solvents on a per-molecule basis, covering a variety of states along the self-assembly process, as shown in Figure 2b as some examples. The definition of assembly energy is the same as in our prior work:^28^

e(AinS)=[E(N·AinM·S)−M·e(S)]/N

where *E*(*N·A* in *M·S*) is the potential energy of *N* polypeptoid molecules immersed in *M* solvent molecules and *M·e*(*S*) is equivalent to the potential energy of the equilibrated solvent-only system. The assembly energy takes into account the peptoid–peptoid, peptoid–solvent, and the intramolecular interaction of each peptoid molecule. For THF/water mixtures, a “unit” consisting of one organic molecule and the corresponding number of water molecules was considered as one virtual molecule (*S*) for the calculation. More discussion about this method is in the Supporting Information and Figure S3. Lower assembly energy represents a more stable nanostructure. In Figure 2a, there is a greater energy drop for THF/water mixture, from isolated molecules to nanofibers and nanosheets, which indicates the favorable self-assembly in this solution, analogous to our prior results for pure water.^28^ By contrast, pure THF shows an energy increase with an increasing number of polypeptoid molecules, prohibiting self-assembly. An exception to this trend is the lower assembly energy for the 6-mer stack in pure THF, implying that small assemblies may be permitted in THF, but no further growth, since the energy cost increases again for larger molecular packings. This is generally consistent with the experimental protocol, where pure organic solvent was used to dissolve synthesized peptoid powders, the self-assembly was initialized by adding a considerable amount of water, and the acetyl or formyl capped Ndc_10_-Nte_10_ finally formed a nanosheet in approximately pure water.^11,18,25^ Compared with pure water, the assembly energy curve shows a larger decrease from finite-width nanofibers to infinitely wide nanosheets in 5% and 4 M THF/water mixture, providing a more significant thermodynamic driving force to form two-dimensional nanosheets. In terms of thermodynamics, the simulation results here indicate the more facile self-assembly of larger nanosheets when there is a small amount of organic solvent.

**Figure 2:**
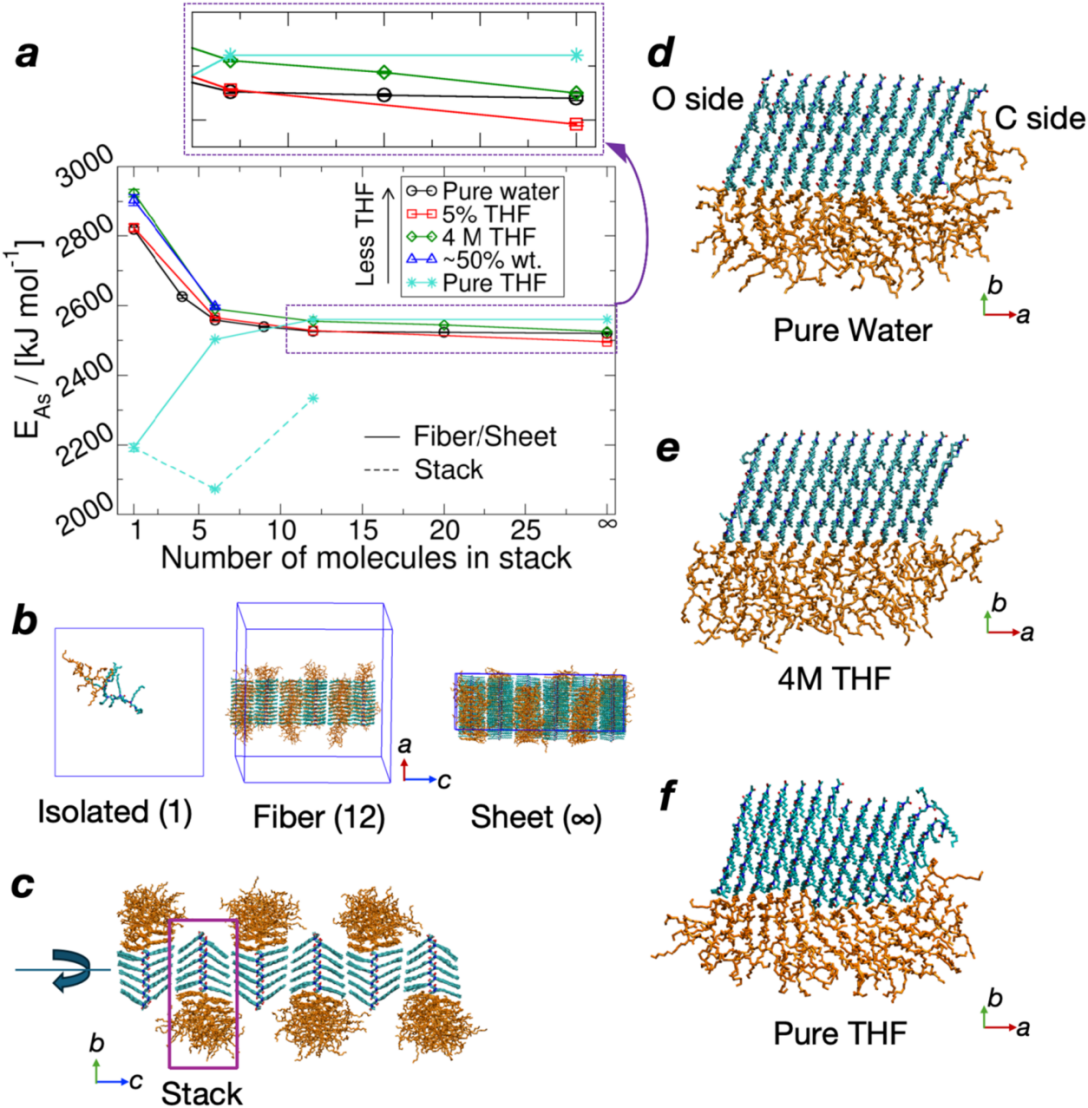
Assembly energy profile and simulated nanostructures of Ac-Ndc_10_-Nte_10_-NH_2_ in different concentrations of THF. (a) Assembly energy profile. (b) Examples of obtained structures for isolated molecules and preassembled nanofibers and nanosheets. (c) Stack in a 12-layer nanofiber. (d–f) One stack from a 12-layer nanofiber in different solvents. In (b–f), cyan: Ndc; orange: Nte. The dashed line in (a) represents a fully solvated single molecular stack. Pure THF cannot form large nanofibers/nanosheets due to the energy cost, while this is allowed in aqueous solutions as the assembly energy decreases. The THF/water mixture has a slightly greater energy drop than pure water from nanofiber to nanosheet. Pure water data were reproduced from ref 28. Copyright 2024 American Chemical Society. Available under a CC-BY 4.0 license.

Visualization of the probed structures shows the stable nanostructures, except for the pure THF case, which exhibited instability, as shown in Figure 2c–f. Although we did not observe that the pure organic solvent could fully dissolve the preassembled nanofiber or nanosheet within finite simulation time, a 12-layer nanofiber in pure THF was observed to disassemble, with a two-molecule block sliding out of the middle of a molecular stack, and the Ndc crystalline blocks are less structured at the end of the stack. These observations are consistent with the thermodynamic assessment of instability and its tendency to dissolve. For the amorphous Nte block, which is invisible in processed cryo-EM data, the presence of organic molecules releases the necessity of coverage of the hydrophobic, crystalline Ndc surface by Nte. We hypothesize that this more open surface should be more accessible for adding more peptoid molecules, increasing the rate of growth of longer nanofibers from the C-side of existing molecular stacks. The presence of more organic molecules at the hydrophobic edges of nanofibers may ease the alignment with neighboring fibers to ultimately form extended 2D nanosheets.

### Water to Assist Aggregation

Self-assembly cannot happen without the aggregation of peptoid molecules. Thus, it is necessary to examine whether the molecular agglomerate can form in an unbiased MD simulation of several dispersed molecules. A box consisting of six separated molecules was simulated to test the possible aggregation, which initially had coiled conformations and were distanced at least 8 Å apart. Figure 3 shows the final structure after the simulation. In pure THF, the polypeptoid molecules remained separated, while agglomeration occurred across the aqueous solutions. This is due to the lower critical micelle concentration in aqueous solutions.^43^ It is notable that with a decreasing ratio of THF, the 6-mer agglomerate has a tighter packing, as measured by the radius of gyration for all peptoid molecules. Therefore, water content not only promotes the formation but also tightens these agglomerates. However, the tightly packed agglomerate might be too stable to have sufficient free space or fluctuation for the subsequent backbone stretching and rearrangement, which is quantified by the negligible standard deviation in the radius of gyration (Figure 3b).

**Figure 3:**
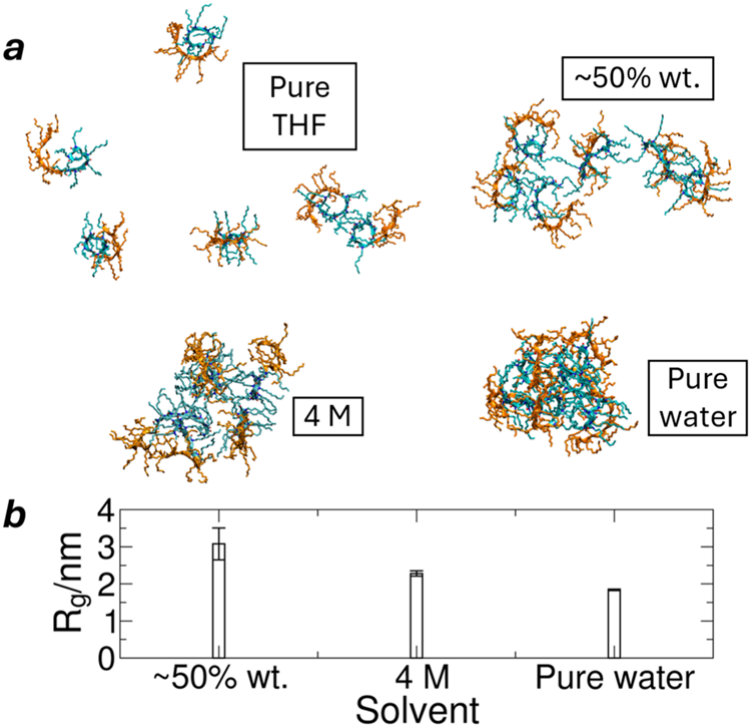
Six-molecule agglomerate in different solvents. (a) Snapshot of polypeptoid molecules after 200 ns of simulation (cyan: Ndc; orange: Nte). (b) Radius of gyration. Pure THF does not permit the aggregation, while an aqueous solvent does. In the THF/water mixture, the agglomerates are loosely packed in comparison to pure water.

### Organic Component to Unfold Backbone

Beyond disordered aggregates, straight backbones are observed for Ndc in the crystalline phase, where the backbone heavy atoms have an all-*cis* conformation and a layer-by-layer alignment, as shown in Figure 2. Hence, it is necessary for a peptoid molecule to open and stretch its coiled backbone in order to slot into or extend nanofibers, and evaluating the chance of backbone unfolding can be useful to investigate the effects of different solvents. As a benchmark, this work used a fully solvated single polypeptoid molecule and implemented a replica exchange with solute tempering technique to enhance the conformational sampling, as described in the Methodology. In water, the molecules are mostly coiled, as shown in Figure 4b. The more hydrophobic Ndc block has a C-shaped curved backbone starting from the N-terminus, which is covered by the less hydrophobic Nte block to reduce its exposure to water. This conformation is significantly different from the straight backbones in the nanocrystals. To quantify the coiling of Ndc, the root-mean-square deviation (RMSD) was calculated for the Ndc backbone atoms with respect to a reference conformation of a C-shape backbone selected from a pure water simulation, as plotted in Figure 4. A smaller RMSD value indicates a more coiled backbone similar to the reference, while a larger RMSD value implies a more open and extended Ndc backbone. It is clear that with increasing THF concentration, the RMSD distribution shifts to higher values, which indicates a more open Ndc backbone conformation. The more open Ndc backbone is consistent with a recent publication, where the hydrophobic segment of a single polypeptoid molecule favored extension into nonpolar organic solvent.^44^

**Figure 4:**
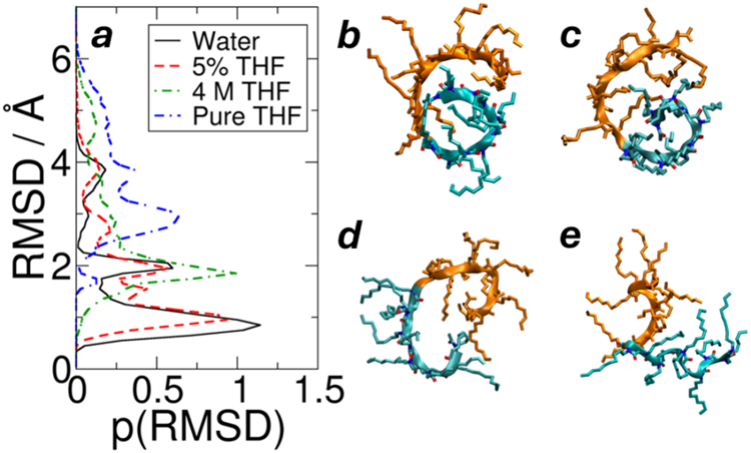
RMSD of the Ndc backbone of an isolated polypeptoid. (a) Histogram of RMSD with a reference of a coiled backbone. (b–e) Representative snapshots of polypeptoid (cyan: Ndc; orange: Nte) in pure water, 5% THF, 4 M THF, and pure THF, respectively. The Ndc backbone of (b) is the reference structure for the curves in (a). Higher THF content opens the coiled Ndc backbone, while the pure water solvent has the most coiled C-shape backbone, as reflected by the peaks at smaller RMSD.

RMSD analysis effectively reduces the structural variation within a large set of long vectors to a one-dimensional distribution through the use of a reference structure from which deviations can be calculated. However, there is the risk that by introducing a reference structure we may bias the analysis and miss other prevalent structures within this complex, multidimensional data set. Therefore, to elucidate whether isolated molecules ever adopt Ndc backbone conformations that resemble those observed in crystalline nanofibers and nanosheets, the UMAP algorithm was employed to reduce the Ndc backbone atomic coordinate information to a two-dimensional map.^39^ Figure 5 shows the plot after UMAP transformation for the isolated molecules, dimers, peripheral C-side molecules in nanofibers, and all molecules in a nanosheet in 4 M THF/water and pure water. Dimers are included because they are the smallest assembly of two aligned polypeptoid molecules. Additionally, C-side molecules from the end of a nanofiber can be slightly curved at the N-terminus, deviating from the straight backbones in the nanofiber interior (Figure S4). These two examples are anticipated as possible intermediate conformations between isolated molecules and crystalline nanosheets. More UMAP results for pure THF and 5% THF/water are provided in Figure S5. One data point on the UMAP plane represents one sampled conformation of the Ndc backbone as observed in the simulations, and close data points correspond to similar conformations in the original (higher-dimensional) atomic coordinates. In 4 M THF/water, the points of some isolated molecules overlap with dimer stacks near those of nanofibers and nanosheets on the UMAP plane in Figure 5a. This indicates that an isolated molecule, assisted by the solvent mixture, can adopt an Ndc backbone similar to those in the crystalline self-assemblies. In contrast, the UMAP data points of the isolated molecules in pure water are quite apart from self-assembled molecules, as shown in Figure 5b, indicating barriers to unfurling the curved backbone in pure water.

**Figure 5:**
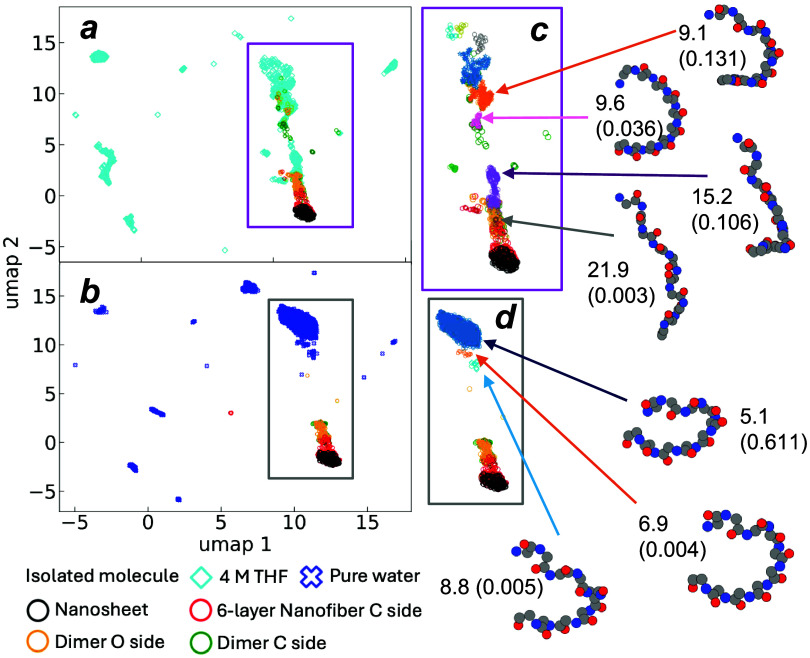
UMAP plot, clustering, and average conformation for isolated molecule, dimer stack, six-layer nanofiber on C-side nanosheet. (a) UMAP of 4 M THF/water; (b) UMAP of pure water; (c) clustering of UMAP data points in 4 M THF/water; (d) clustering of UMAP data points in pure water. The numbers near the averaged conformation are the end-to-end distance and its population in parentheses. With some THF, an isolated molecule presents some conformations overlapping with the dimers, indicating the possible backbone unfolding. In pure water, conformation of an isolated molecule is apart from those in nanostructures, which implies the difficulty of backbone unfolding and the subsequent nanocrystal growth.

To further identify the promising conformations with straight Ndc backbones, the HDBSCAN algorithm was used to cluster all data points of isolated molecules,^40^ which grouped the similar backbone conformations to one cluster on the UMAP plot. HDBSCAN filters out scattered noise points and only groups the points in the dense regions of the sampled data, which is a useful method to extract insights from MD simulations.^45^ Note that here the word “cluster” is used to indicate similarity in data and does not imply the aggregation or agglomeration of molecules in solution. Points on the UMAP plane within the same cluster are expected to adopt similar conformations. We pay particular attention to the clusters of points in the UMAP plane near those of the nanosheets, nanofibers, and dimers, as these molecules before self-assembly are expected to have conformations resembling the molecules after self-assembly. Figure 5c shows the clustering of data from isolated molecules that are deemed similar to that from the self-assembled structures. Distinct clusters are indicated by recoloring data points from the outlined region of Figure 5a. The average Ndc backbone conformations of these clusters are shown, along with their end-to-end distance and population proportion. There is a small population (∼0.3%) of very open backbones having an average end-to-end distance of about 21.9 Å, and a considerable population of nearly opened (10.6%) with an average end-to-end distance of 15.2 Å, approaching the 26 Å of maximally extended Ndc backbones in nanosheets. Semiopen C-shaped backbones are also observed with an average end-to-end distance of ∼9 Å in 4 M THF, some of which have a perfect *cis* conformation as observed in nanofibers and nanosheets. These are considered as promising conformations for self-assembly. On the other hand, in pure water, HDBSCAN clustering analysis of select data in Figure 5d indicates that there are only coiled backbones with smaller end-to-end distances, which reflects the difficulty of backbone opening without THF. Although this analysis only includes the sampling of isolated molecules instead of peptoid agglomerates, the comparison between pure and mixed solvent implies that backbone coil opening and extension is assisted by the organic solvent, which may indicate its importance in accelerating the assembly of crystalline Ndc in nanosheets, despite its primary role in dissolving these molecules initially.

### Preferential Solvation on Hydrophobic Blocks

As discussed above, the organic component of the solvent mixture assists the backbone opening, and thus, it is worth knowing the exact local solvation of the peptoid surface, especially near the end of the self-assembly process. Figure 6 shows various details of the local molecular ratio of THF/water as a function of proximity to a self-assembled peptoid sheet for an overall concentration of 4 M THF/water. In Figure 6b, it is shown that the organic molecule has a 3–4 times higher concentration near the peptoid surface. The highest concentration of THF is found near the decyl side chains, as shown in Figure 6a, and the concentration gradually decreases when it comes to the amorphous Nte blocks but is still higher than the bulk solvent far from the peptoid nanostructure. Previous experimental measurements have not explored such preferential solvation of peptoids, and stated concentrations were simply estimates based on mixing ratios.^18^ Figure 6c and d visualize distinct differences in solvent coverage for THF vs water near Ndc and Nte blocks, respectively. THF covers the Ndc side chains and is embedded in the amorphous Nte, while water covers the acetyl N-terminus and Nte but is rarely found near the Ndc decyl side chains. This reflects the significant difference in hydrophobicity between segments of the proscribed molecular sequence of this designed and synthesized peptoid. As a result, a very low concentration of THF could have a sufficient effect as a mixed solvent. A small amount of THF in water would enhance the self-assembly more than a homogeneous THF/water mixture of the same concentration. It is worth noting that this preferential solvation is also evident before the self-assembly, where there are more THF molecules and rare water near Ndc for an isolated polypeptoid molecule (Figure S6). Recalling that Ndc is the block forming crystalline phase whose backbone opening/extension is assisted by THF, this selective solvation likely contributes to the facile formation of these highly ordered nanostructures.

**Figure 6:**
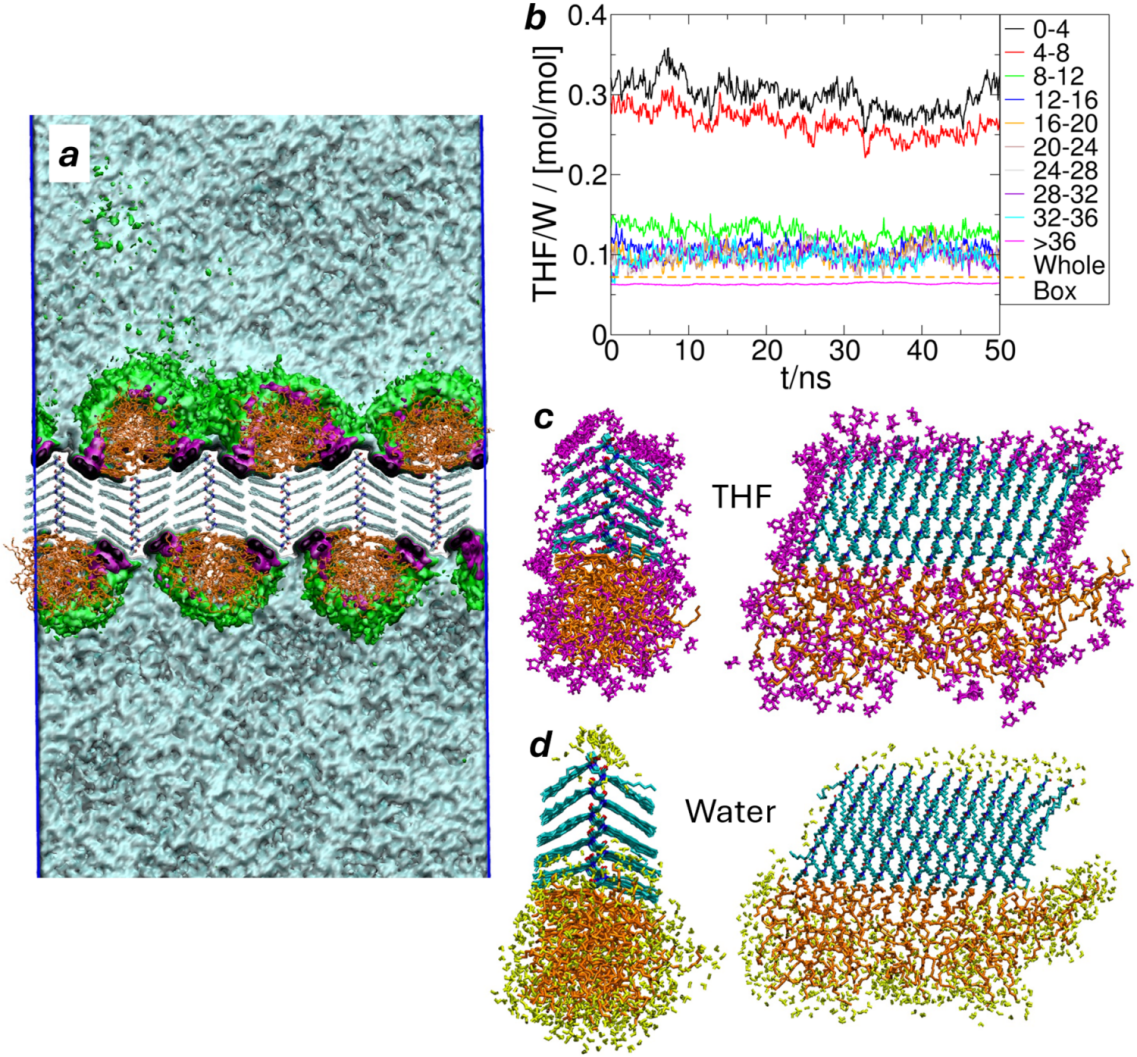
THF coverage of peptoid nanocrystal. (a) Density probability map of THF near the nanosheet, indicating distinct domains as black: 0.5; purple: 0.3; green: 0.2; and cyan: 0.1 g/cm^3^. (b) Molar ratio at distances from the peptoid surface. (c) THF within 4 Å of one stack forming a 12-layer nanofiber; (d) water within 4 Å of one stack forming a 12-layer nanofiber. In (c) and (d), cyan: Ndc; orange: Nte; magenta: THF; yellow: water.

### Surface Adsorption of Organic Solvent

The high surface concentration of THF indicates specific adsorption of THF by the polypeptoid. Therefore, evaporation-induced self-assembly can be ultimately regarded as the removal of organic solvent from the peptoid surface (and not just from the bulk solution). We attempted to evaluate the free energy cost to complete such a removal. In Figure 7, umbrella sampling was used to monitor the free energy when displacing a THF molecule from an existing nanosheet surface. Based on the profile in Figure 6a, the starting point was chosen as the adsorbed THF on the decyl side chain of Ndc. It was gradually pulled from this region to the bulk solvent by increasing its minimum distance to the nearest Ndc atom. Figure 7c plots the regions where the THF molecule is most likely to reside when it is held at a certain distance from the nearest Ndc atom. The contours show successful coverage of all representative locations at each stage. Accordingly, the desorption path can be described as follows: the THF molecule first leaves the decyl side chains to settle in the amorphous Nte and finally dissociates from Nte to fully dissolve into the bulk solvent. Figure 7a shows the free energy cost to desorb a THF molecule in a 4 M THF/water mixture. The highest free energy barrier is for THF to desorb from the decyl side chains. The subsequent barriers at larger distances are due to the THF concentration change and the contact with the Nte ether side chains. The overall cost of desorption is 2.5–4.5 kJ/mol, which is close to 1 *k*_B_ *T* (2.5 kJ/mol) at 300 K. Figure 7b, as a comparison to Figure 7a, shows higher barriers and overall cost to remove THF in pure water, which correspond to the extreme condition of complete evaporation of THF. In each solvent, the high variance for the three independent runs can be attributed to the fluctuating desorption path, but this does not affect the comparison between Figure 7a and b. This result implies that the desorption becomes much more difficult at the end of the evaporation, and thus, the complete removal of organic solvent is hard to reach. It is consistent with the experiments, which reported incomplete THF removal after the evaporation-induced self-assembly.^18,25^ In the experiment, we propose that the entire self-assembly process actually occurs in a mixture of abundant water with a small amount of THF on the peptoid surface. As indicated above, this remnant fraction of THF may indeed facilitate the assembly process by reducing barriers to the formation of extended Ndc backbones for the crystalline region, by increasing the available free space for conformational changes overall by permeating the Nte blocks, and perhaps by performing some lubricant function for the alignment of assembled molecular stacks to form extended fibers and sheets during growth.

**Figure 7:**
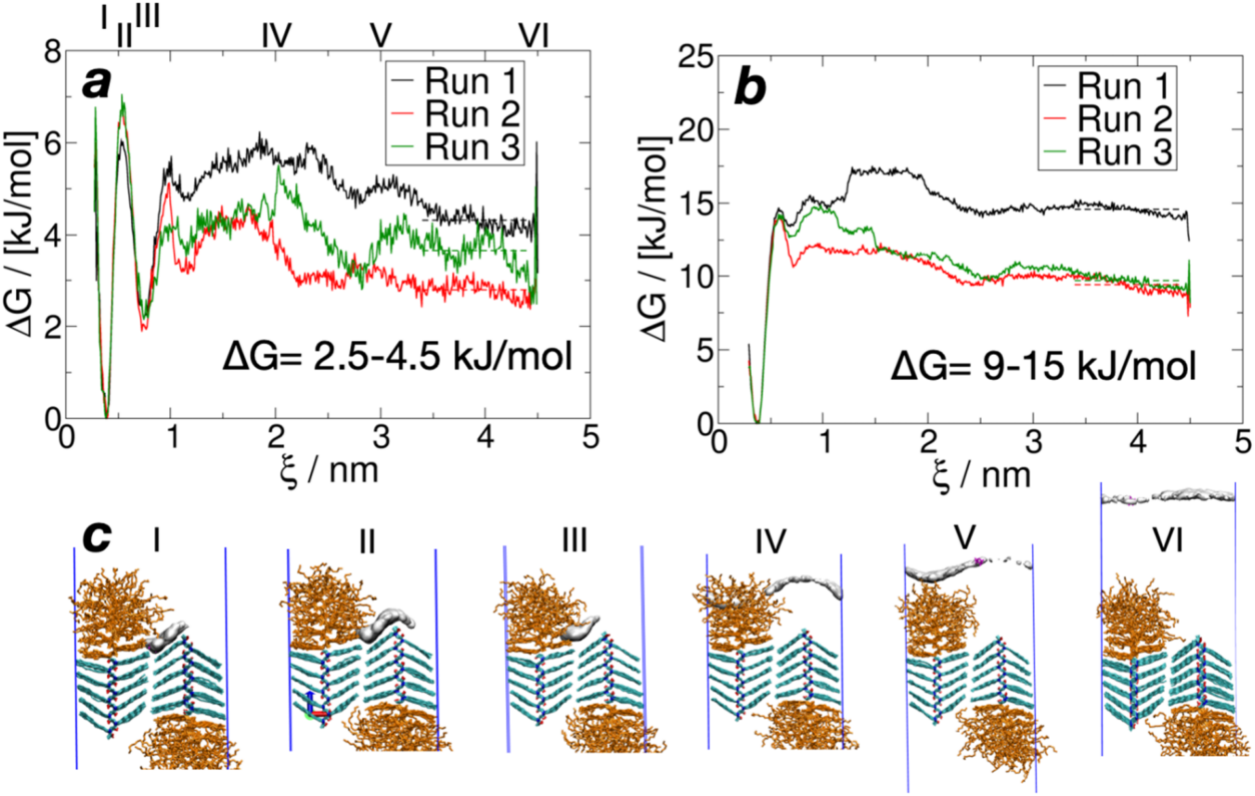
Free energy of surface desorption. (a) Potential of mean force for 4 M THF/water; (b) for pure water (only one THF molecule); (c) sampled regions for 4 M THF/water (cyan: Ndc; orange: Nte), showing the contours of 0.0 g/cm^3^ for density probability. The approximate position of each sampling window is labeled on top of (a). The energy barriers are higher with a lower THF concentration, indicating the more difficult removal of THF from the peptoid surface at the end of the evaporation-induced self-assembly.

## Conclusions

Using MD simulations and enhanced sampling techniques, this work has demonstrated the roles of organic molecules and water in the self-assembly of an Ac-Ndc_10_-Nte_10_ diblock polypeptoid. By simulating the isolated molecules and preassembled nanofibers and nanosheets, the assembly energies provide a comprehensive evaluation of their relative stability, indicating the thermodynamic driving forces of self-assembly. We observe that self-assembly to form extended peptoid nanostructures is unfavorable in pure THF due to an associated energy cost but is facilitated in aqueous solutions, consistent with experimental observation. Our findings indicate that tuning the solvent mixture may advance the driving force to form extended 2D nanosheets, which we observe to be slightly enhanced over assembly in pure water. We posit that at the molecular level, this assembly process relies on the simultaneous presence of both water and THF. As confirmed by MD simulations, more water promotes the aggregation of polypeptoid molecules, corresponding to the decrease of the critical micelle concentration for crystallization by diluting the THF content. On the other hand, THF maintains a loose agglomerate and promotes the unfolding of the Ndc backbone, as indicated by the radius of gyration for polypeptoid agglomerates and the analysis of conformations for different assembly stages. Since the crystalline phase has extended and well-aligned Ndc backbones, the conformation change and the backbone–backbone alignment are necessary for an isolated molecule to become a nanocrystal molecule. Hence, some THF is beneficial not only for tuning the solubility but also for controlling the polypeptoid conformation. Prospectively, we expect that other organic solvents could similarly facilitate the self-assembly of polypeptoid into nanosheets with highly ordered internal structures, provided they are carefully chosen as good solvents for polypeptoid. For polypeptoids, we propose a cooperative self-assembly process, where both water and organic components have their own roles.

Considering the very slow evaporation of the organic solvent in the experiments, we propose that the self-assembly occurs in a water/organic mixture; that is, nanocrystals have formed even when a small amount of organic solvent remains. Our simulations have demonstrated the strong adsorption of THF to the peptoid surface, maintaining a significantly higher relative concentration when equilibrated with the bulk solution. Specifically, THF prefers the more hydrophobic Ndc block, which is the component of the crystalline phase; less water is present in this region. Moreover, the desorption of THF into the bulk solution becomes more difficult as the overall concentration reduces, implying that it may persist at the surface of peptoids for long times, despite evaporation from the bulk solution. Although past experiments have not intentionally maintained the amount of the organic solvent nor measured its surface concentration, our simulations indicate that maintaining a solvent mixture may be beneficial for the preparation of large polypeptoid nanocrystals in this case.

## Supplementary Material



## Notes

Coiled backbone in pure water; UMAP and HDBSCAN workflow; discussion of the method to calculate assembly energy; dimer and peripheral molecules on C-side; UMAP plots for 5% THF/water and pure THF; visualization of an isolated molecule solvated in 4 M THF/water (PDF)
