# Supplementary material for: Cooperative Role of Mixed Solvent in the Evaporation-Induced Self-Assembly of Polypeptoid Nanocrystals
Source: ACS Appl Nano Mater. 2025 Jun 16;8(25):12909–19. doi: 10.1021/acsanm.5c01381 (PMC12210217; doi:10.1021/acsanm.5c01381)
Supplement: Supplementary file 1 [file an5c01381_si_001.pdf]

Supporting Information

Cooperative Role of Mixed Solvent in the Evaporation-Induced Self-Assembly of Polypeptoid Nanocrystals

Xubo Luo[1], Fabrice Roncoroni[2], Tianyi Yu[1], Nan K. Li[1], Ronald N. Zuckermann[1,2], Xi Jiang[1], Nitash P. Balsara[1,3], David Prendergast[1,2]*

[1] Materials Sciences Division, Lawrence Berkeley National Laboratory, Berkeley, CA 94720, USA

[2] The Molecular Foundry, Lawrence Berkeley National Laboratory, Berkeley, CA 94720, USA

[3] Department of Chemical and Biomolecular Engineering, University of California, Berkeley, CA 94720, USA

[*] Corresponding author: David Prendergast, dgprendergast@lbl.gov

Assembly energy. The calculation of assembly energy assumed that the bulk solvent mixture, the concentration of which was solely determined by counting the molecules, remained the same before and after adding peptoid molecules. The potential energy of the solvent mixture was calculated from a solvent-only box, which was then subtracted from the total potential energy of a peptoid-solvent box. In a later discussion of the main text, it shows that this may introduce some numerical inaccuracy. THF may be more attached to the peptoid surface and, for fixed numbers of molecules, this would slightly reduce its effective bulk concentration. This begs the question: whether our assembly energy is affected by this non-uniform solvent concentration. To verify, we conducted a test using larger simulation boxes, with more solvent molecules. Thus, the surface adsorption of THF should have a relatively smaller effect on the bulk solvent concentration. The red and green curves in Figure S2 show that there is some tiny variance, provided that each solvent component had sufficient molecules at the investigated concentration. In this situation, the surface adsorption does not affect the comparison of solvent mixture and pure water, and the enhanced tendency to form nanosheets in THF/water mixture remains the same as discussed in the main text. However, we admit that this approach is not applicable for trace concentrations of either component. We attempted testing simulations of trace amounts of water in THF, i.e., only one or two water molecules in a box with one peptoid solvated predominantly by THF, labeled as '1W' and '2W' in Figure S2. There is a sudden energy jump for an isolated molecule, which could be due to the finite box and the 'cold' setting of solvent molecules in REST2 simulation. There would be some uncertainty in estimating the bulk concentration with the presence of peptoid. As a result, our method of assembly energy is not suitable, in this trace limit, to obtain the exact concentration dependence of the assembly energy curve transition (switching from energy cost to energy gain). However, based on the switch from stable dissolved peptoids in pure THF to unstable with only 2 water molecules, we can at least say that this switch occurs at low water concentrations.

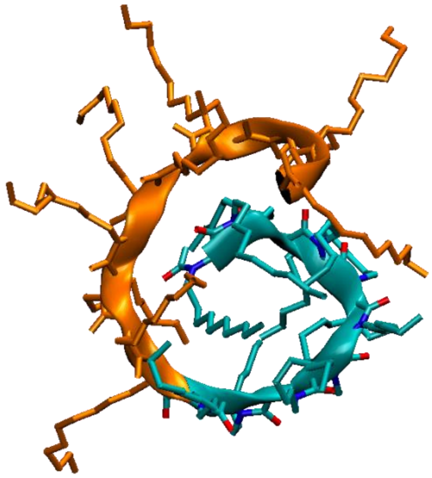

Figure S1. Coiled backbone of an isolated Ac-Ndc_{10}-Nte_{10} in pure water.[1] The hydrophobic Ndc10 block is shown in cyan. The more hydrophilic Nte10 block is shown in orange. Peptoid backbone moieties are indicated specifically (amide N in blue, carbonyl O in red).

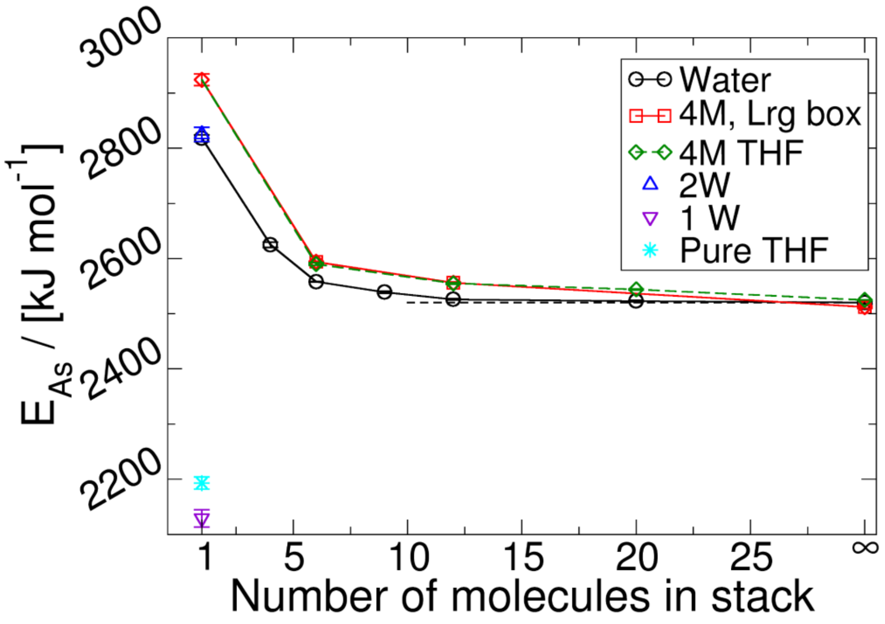

Figure S2. Energetics comparison of two different box sizes at the concentration of 4 M THF/water and tiny amounts of water.

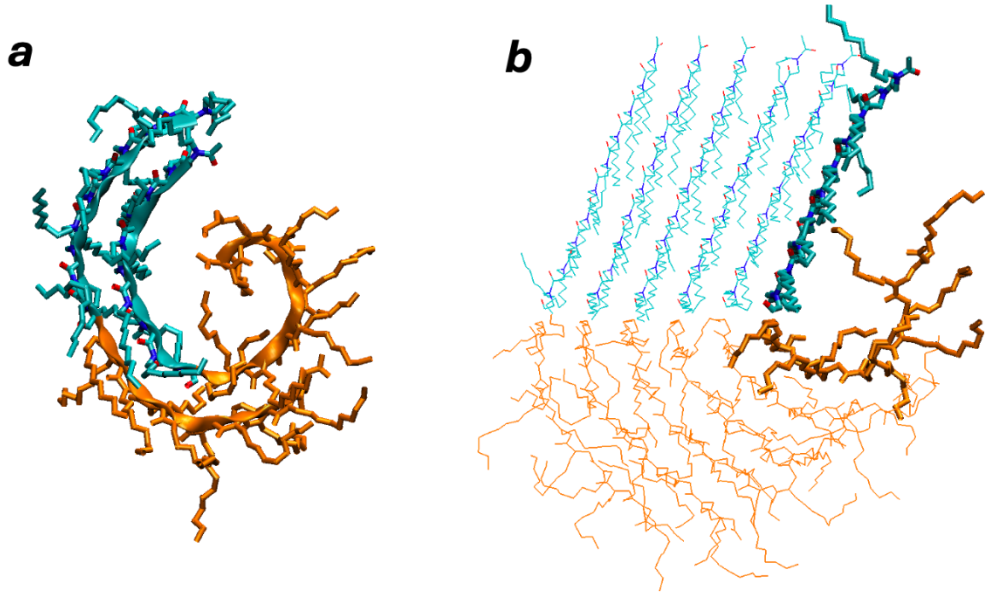

Figure S3. Dimer and peripheral terminal C-side molecule in 4 M THF/water.

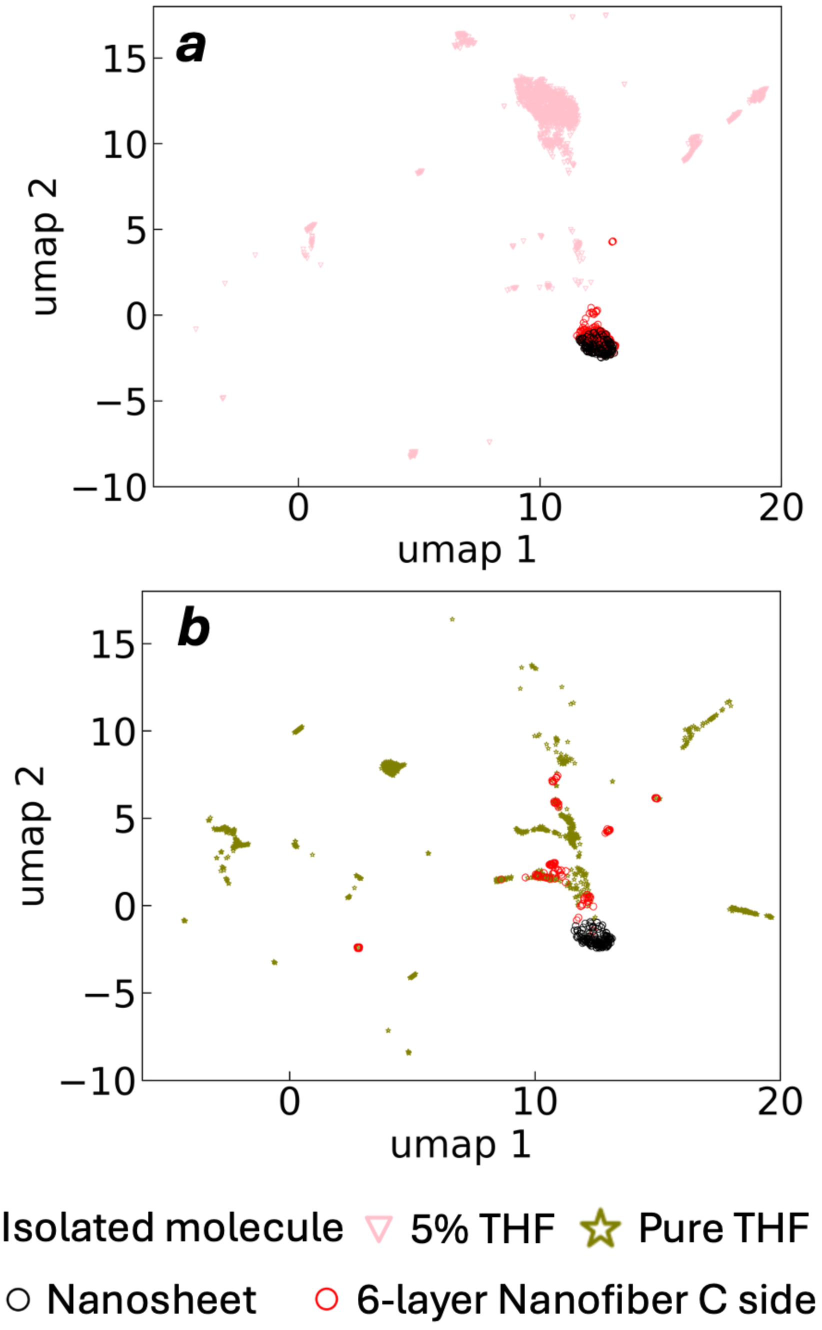

Figure S4. UMAP analysis for (a) 5% THF and (b) pure THF. Isolated molecules (pink inverted triangles and olive stars for 5% THF and pure THF, respectively), C-side terminal molecules (red circles) and nanosheet molecules (black circles) are plotted. Lower concentration of THF in (a) has fewer points representing isolated molecules near the black points of nanosheets. Pure THF shows more isolated molecule points near black nanosheet points.

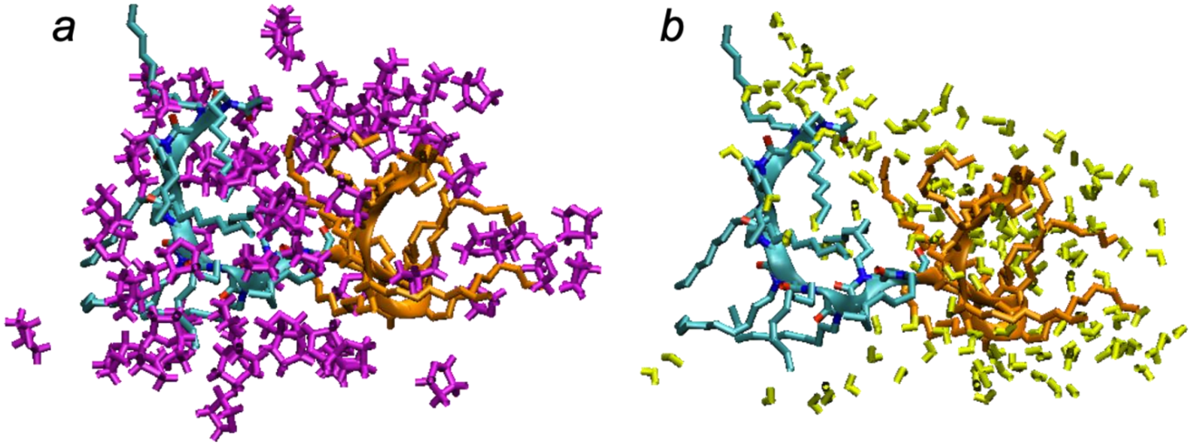

Figure S5. Solvent separation at the monomer surfaces in 4 M THF/water solvent. a. THF molecules (magenta) within 4 Å of peptoid; b. Water molecules (yellow) within 4 Å of peptoid. Molecular-scale phase separation is observed before self-assembly.

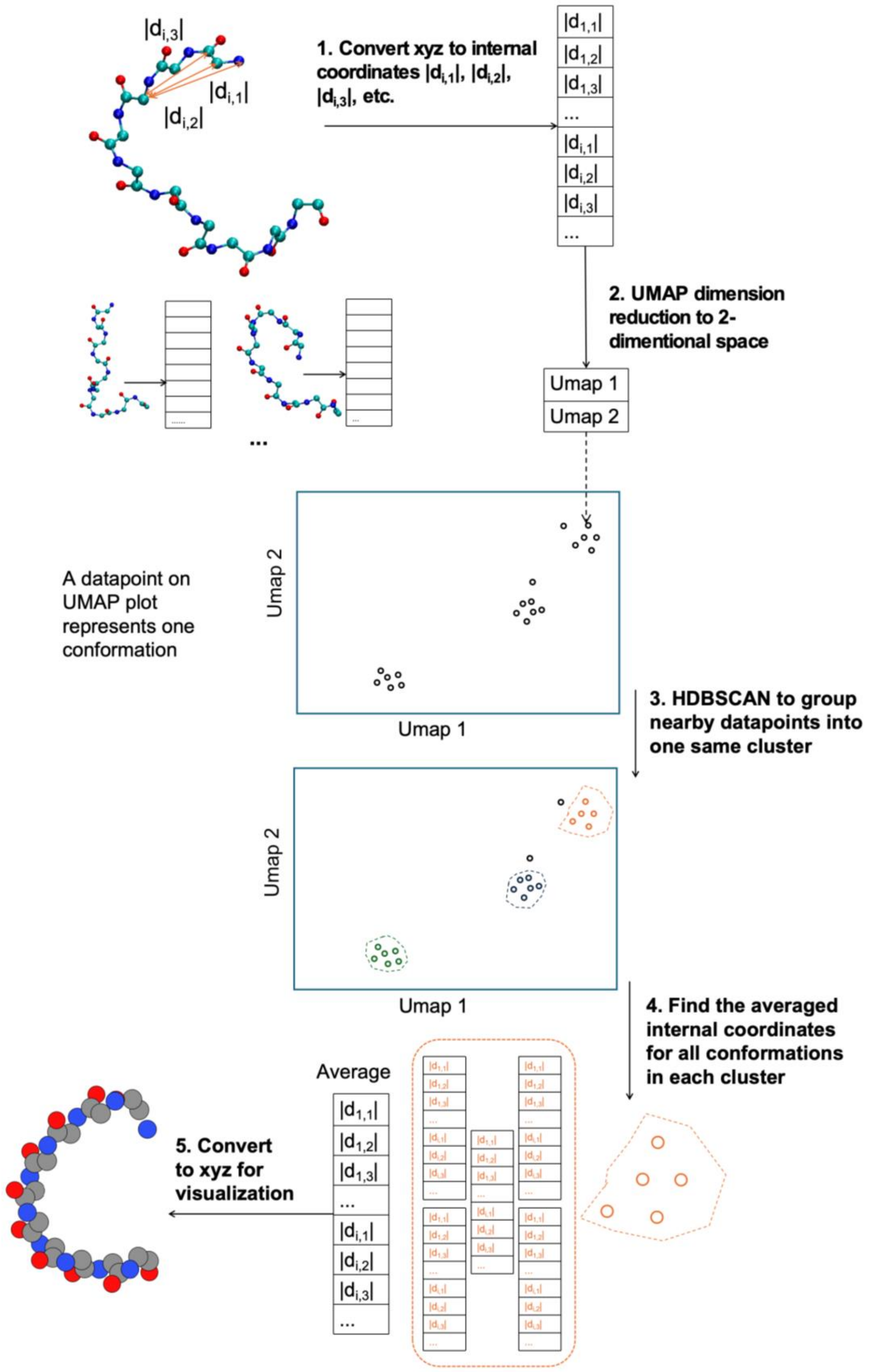

Figure S6. UMAP and HDBSCAN workflow.

Reference:

(1) Luo, X.; Yu, T.; Li, N. K.; Zuckermann, R. N.; Jiang, X.; Balsara, N. P.; Prendergast, D. Thermodynamic Driving Forces for the Self-Assembly of Diblock Polypeptoids. *ACS Nano* 2024, *18* (23), 14917–14924. https://doi.org/10.1021/acsnano.3c12228.
